# Shared decision-making and patient decision aids in knee osteoarthritis: a scoping review

**DOI:** 10.1186/s43019-026-00310-y

**Published:** 2026-02-23

**Authors:** Ethan Wang, Amber Stefanski, Tsz King Donald Chow, Angela Spencer, Keniesha Thompson, Sheyna Gifford

**Affiliations:** Saint Louis University School of Medicine, 1402 S Grand Blvd, St. Louis, MO 63104 USA

**Keywords:** Knee osteoarthritis, Shared decision-making, Patient decision aids, Scoping review

## Abstract

**Background:**

Shared decision-making is a collaborative approach that enables clinicians and patients to make informed treatment decisions that align with patient preferences. This study examined current practices of shared decision-making in managing knee osteoarthritis.

**Methods:**

A comprehensive search was conducted in Cochrane, CINAHL, ProQuest, Scopus, Ovid MEDLINE, and Web of Science databases through August 2025. EndNote 21 was utilized for de-duplication, and Rayyan was used for screening. Studies were analyzed for the use of patient decision aids, shared decision-making outcome measures, and barriers and factors impacting shared decision-making practices.

**Results:**

Of 4708 records screened, 69 studies were included for analysis, comprising 23 randomized controlled trials, 18 qualitative studies, 9 trial protocols, and 19 other observational designs. A total of 44 studies explicitly described the use of a patient decision aid. Reported outcome domains included decision quality, decisional conflict, satisfaction, regret, and patient–clinician communication. Commonly cited barriers to shared decision-making (SDM) implementation were limited clinician time, lack of awareness of patient decision aids (PDAs), and patient difficulty using digital tools.

**Conclusions:**

The included studies suggest that patient decision aids may be a valuable tool for management of knee osteoarthritis, with studies reporting improvements in patient engagement and informed decision-making. However, variability in shared decision-making implementation and inconsistency in outcome measures highlight the need for further research to evaluate the comparative effectiveness of decision aids in clinical practice.

**Supplementary Information:**

The online version contains supplementary material available at 10.1186/s43019-026-00310-y.

## Background

Knee osteoarthritis (OA) is a prevalent and debilitating musculoskeletal condition characterized by progressive articular cartilage degeneration and structural modifications in the surrounding bone and tissue structure [1]. The process involved in these pathological changes is mediated by a complex interplay of inflammatory and growth-signaling molecules that have yet to be fully understood. Among the elderly population, knee OA is the leading cause of chronic disability, with symptomatic knee OA occurring in 10% of men and 13% of women aged 60 years or older [2]. Apart from age, risk factors such as gender, obesity, occupation, knee alignment, or previous surgeries also play a role in the pathogenesis and progression of knee OA [2]. Given its complexity, knee OA management presents substantial functional and economic burden on patients.

The management of knee OA typically involves a multidisciplinary approach, incorporating lifestyle modifications, pharmaceutical interventions, and surgical procedures. The main goal of treatment includes reducing pain and inflammation while maintaining or improving range of motion, function, and overall quality of life [3]. However, applying these guidelines universally remains challenging owing to individual factors such as comorbidities, knowledge gaps, and poor medical adherence. For example, patients who are sedentary with knee OA may encounter more difficulties in maintaining physical and dietary behavioral changes, leading to worsened pain and quality of life [4]. Furthermore, elderly patients often struggle to make informed treatment decisions owing to the uncertainty of disease progression and inadequate knowledge about knee OA. This makes evaluating and predicting disease consequences more challenging, resulting in additional stress and worsened decisional outcomes [5].

Shared decision-making (SDM) is a collaborative process in which patients and healthcare providers evaluate and communicate preferences regarding different care options. It is a critical aspect of patient-centered healthcare as it enables open communication between clinicians and patients about treatment options on the basis of clinical evidence and patient values [6]. The principle behind SDM is well described among several structured models. One widely used framework is Elwyn’s three talk model, which provides a practical approach to fostering a collaborative decision-making process with patients through three sequential steps: team talk, option talk, and decision talk. Multiple studies have shown that implementing SDM in clinical settings can promote active decision-making and improve patients’ risk perception [7]. SDM also effectively reduces decisional conflicts, which are especially important when patients are facing treatment decisions involving high uncertainty or risks [8]. In recent years, SDM has been implemented through patient decision aids (PDAs), which are booklets, interactive programs, or websites designed to help patients make informed decisions [9]. Several PDAs have been developed in clinical practices for knee OA to encourage a shift from physician-controlled decision-making to patients taking a more active role in decision-making [10].

**Fig. 1:**
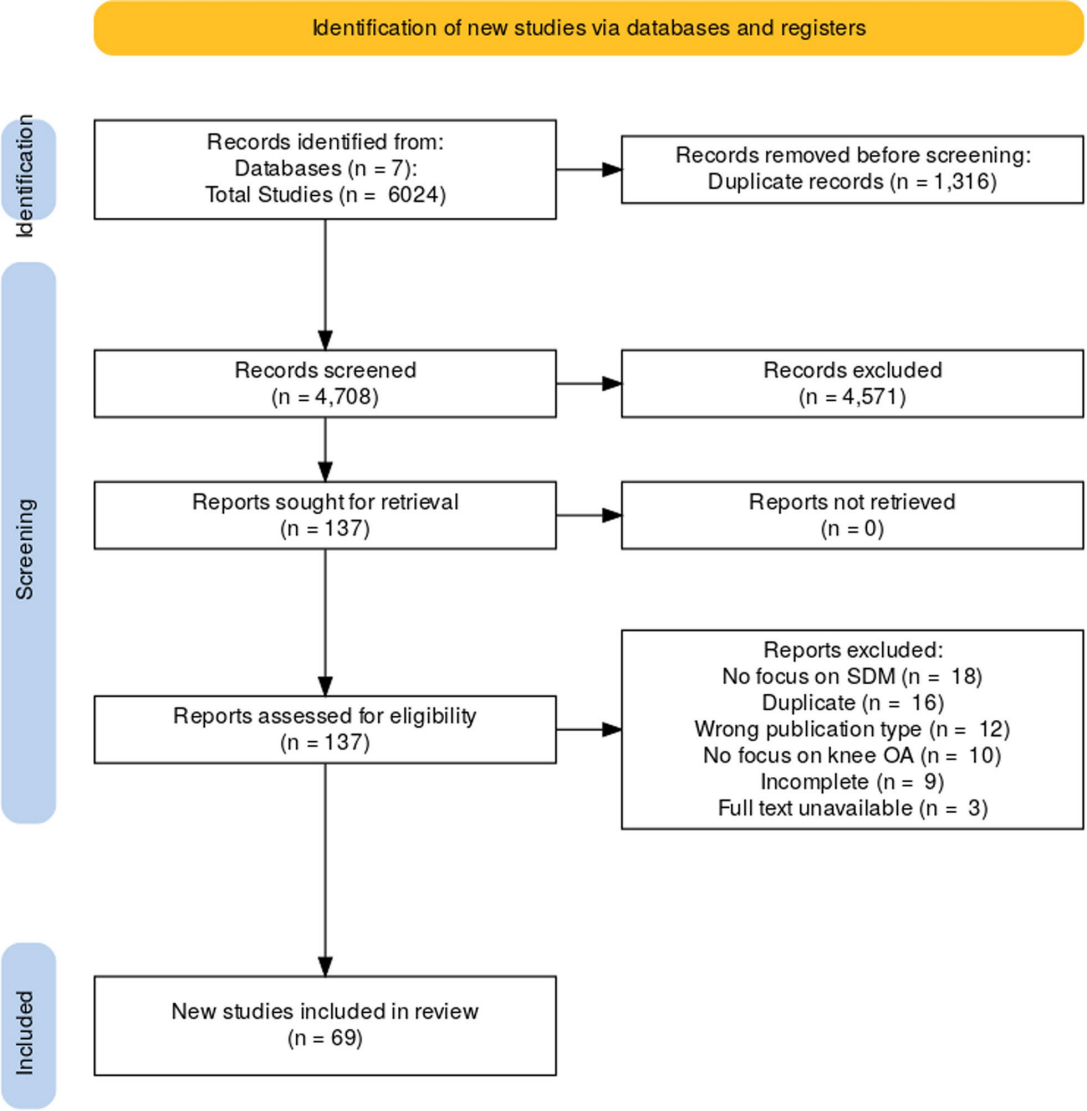
Preferred Reporting Items for Systematic Reviews and Meta-Analyses (PRISMA) flow diagram

The management of knee OA presents several opportunities for integrating SDM. Previous research has shown that patients with knee OA often face challenges owing to a lack of knowledge about conservative treatments and insufficient guidance from healthcare providers [11]. The abundance of information and the limited clinic consultations can further compound these difficulties. Therefore, the collaborative approach of SDM enables patients to navigate the expanding array of treatment options more effectively while maintaining evidence-based knowledge in light of the unpredictable nature of their diseases. Multiple studies have examined the use of PDAs in patients with knee OA, highlighting their positive impact on patient knowledge, reduction in decisional conflict, or increased readiness to make decisions on the appropriate treatments [12].

Our study aims to investigate the existing practices of SDM among patients experiencing knee OA. We utilized a scoping review methodology, which involved synthesizing research findings and identifying research gaps on a specific topic to guide practice, policy, and future research. By understanding and shedding light on the current SDM practices related to knee OA, we hope to encourage further research on the utility of SDM in the knee OA patient population that can ultimately promote improved patient health outcomes.

## Methods

### Search strategy

We conducted a scoping review in accordance with the methodological framework developed by Arksey and O’Malley [13] and the PRISMA extension for scoping reviews [14]. The objective of this review was to assess the current state of SDM literature pertaining to patients affected by knee OA. Initial searches were conducted between 26 January 2025 and 14 February 2025, and a subsequent search was performed with the same query on 8 August 2025, to capture articles published after the initial search. The following electronic databases were utilized: Cochrane Database of Systematic Reviews (Wiley), Cochrane Central Register of Controlled Trials (Wiley), CINAHL (Ebsco), MEDLINE (Ovid), Dissertations & Theses (Proquest), Scopus (Elsevier), and Web of Science (Core Collection-Clarivate). Searches were also carried out in PEDRO and RehabDATA, but no additional articles were found that had not been discovered by the other databases. The databases chosen were selected on the basis of the desire to capture a multitude of areas in which knee OA, patient-centered care, and SDM may be discussed. Each database was searched for the following terms using both Boolean and proximity operators, keywords, and Medical Subject Headings (MeSH): knee osteoarthritis, (knee AND osteoarthritis), decision making, shared decision making, decision aid*, with other variances. No filters or limits were added to the searches. Results were included regardless of date, article type, or subject. Our only exclusion criteria for the initial search was if an English version was unavailable. Database searches yielded a total of 6024 results. EndNote 21 was employed for de-duplication, resulting in the exclusion of 1316 duplicates.

### Article screening

All results were then imported into Rayyan, a tool for systematically reviewing articles. A total of 4708 articles were screened. Our inclusion criteria for initial screening was broadly that the references discussed SDM in a patient population that included patients with knee OA. Two reviewers (D.C. and A.L.S.) title- or abstract-screened all articles for inclusion criteria. Excluded references were studies irrelevant to either knee OA or SDM, studies that were not available in English, opinion or magazine articles, incomplete studies, or those that did not have a full-text version available. Both reviewers were blinded to the decision of the other throughout the screening. If there was a discrepancy between whether to include or exclude a reference, a third reviewer (E.W.) resolved it.

Several studies enrolled patients with knee OA and OA of other joints. To prioritize capturing all relevant knee OA patient data, these studies were retained and results extracted as reported, even if they included mixed-joint populations. When multiple publications were suspected to originate from the same cohort, the most comprehensive or recent report was retained to minimize duplication; however, some overlap remains possible owing to variations in reporting descriptions. Protocol papers were included for completeness to capture the breadth of ongoing research; however, only corresponding published results were analyzed in the discussion.

### Data extraction

Following title and abstract screening, 137 articles remained for full-text consideration. Each reference was reviewed for inclusion by at least two reviewers (D.C., A.L.S., or E.W.). The third reviewer acted as a tie-breaker when necessary. After full texts were reviewed, 69 references were included for final analysis and discussion (Fig. 1). The references included were then coded for whether or not they discussed a specific PDA; provided a definition of SDM; discussed positive, negative, or null results of implementing SDM in practice; or discussed attitudes toward SDM, factors affecting the implementation of SDM, or strategies for implementing SDM.

## Results

Out of 4708 studies screened, 69 were included in the analysis (Appendix 1: Included Studies) [15–83]. The included studies were coded according to the predefined framework described above (see Appendix 2 for the detailed mapping of studies to shared decision-making components and outcome domains). Of the 69 included studies, 37 enrolled patients with knee osteoarthritis only, while 32 included mixed-joint osteoarthritis populations involving both the knee and hip. These 69 studies comprised 23 randomized controlled trials, 18 qualitative studies, 9 randomized controlled trial protocols, 8 cohort studies, 7 cross-sectional surveys, 1 PDA validation study, 1 mixed-methods study, 1 pre–post study, and 1 participatory action research study. Of these, 35 studies (51%) were conducted in the USA, while 34 were from other countries. The 69 SDM studies on knee OA involved a total of 75,631 patients and 156 clinicians.

### Patient decision aids

Of the 69 studies included for analysis, 44 studies explicitly describe or mention use of a PDA to facilitate shared decision-making. There was considerable variability in how thoroughly each tool was described within each study; many tools are not free and publicly accessible, and many studies did not provide a name for their tool. Therefore, it is possible that some studies utilized the same tool, but we were unable to confirm this solely on the basis of the descriptions provided in the articles. When it was possible to verify that multiple studies discussed the same tool, the studies were listed together in Table 1. In general, most tools aimed to provide information on the disease process of knee OA as well as various surgical and nonsurgical treatment options. Some studies employed tools that focused solely on nonsurgical options [27, 33, 37, 47, 63, 65, 66, 73, 77].

**Table 1:** Summary of patient decision aids in included publications

Source(s)	Name of decision aid (developer)	Format	Purpose or description
Allen et al. (2016); Arterburn et al. (2012); Bozic et al. (2013)	Name not specified (Informed Medical Decisions Foundation and Health Dialog, USA)	44-min video and printed booklet	To inform patients with knee OA of various treatments, including behavioral, rehabilitative, pharmacological, and surgical options
Bansback et al. (2022)	Name not specified	Online, PROM-based patient decision aid	To educate patients with knee OA regarding both surgical (total knee replacement [TKR]) and nonsurgical treatment options. Compares treatment options on the basis of the chance of repeat surgery, need for physical therapy, recovery period, and quality of life changes
Boland et al. (2018); Mangla et al. (2018, 2019); Sepucha et al. (2017); Shue et al. (2016); Stacey et al. (2014, 2016); Trenaman et al. (2017)	Treatment choices for knee OA (Health Dialog, USA, 2014)	42-min video and 38-page printed booklet	To discuss the concept of SDM, the pathophysiology of arthritis, and available treatment options (both surgical and nonsurgical) for patients with knee OA
Bossen et al. (2024)	Name not specified	Online	To provide information, comparison of treatment options, a summary of important points, patient preferences, and questions to verify patient knowledge for patients with knee OA
Brodney et al. (2022); Sepucha et al. (2019, 2021)	Knee osteoarthritis: is it time to think about surgery? (Healthwise, 2016)	An online, interactive tool or a printed 12-page brochure	To provide details on nonsurgical treatment options (e.g., lifestyle changes, physical therapy, walking aids, pain medications, injections, complementary approaches) as well as surgical options (TKR and partial knee replacement [KR]) for patients with knee OA
Churchill et al. (2022)	Name not specified	Five videos	To optimize nonoperative management and decision-making in patients considering TKR for knee OA. Videos explained knee anatomy and disease process, appropriate imaging for diagnosis, nonoperative treatment options, indications for TKR, and surgery expectations
Daudelin et al. (2020)	Knee Osteoarthritis Mathematical Equipoise Tool	Online	To provide patient-specific decision support for patients with knee OA, considering both nonsurgical treatments and surgical TKR
De Jesus et al. (2017)	Name not specified	Print	To educate patients with isolated medial compartment knee OA on the benefits and risks associated with unilateral and total knee arthroplasty in terms of recovery, functional results, and revision rates
Dolan et al. (2013)	Name not specified	Computerized dashboard	To summarize information about the effectiveness, risks, costs, and ease of use associated with nine analgesic treatment options for patients with knee OA
Elwyn et al. (2016); Kinsey et al. (2017); Wood et al. (2017); Reilly et al. (2023)	The knee osteoarthritis Option Grid (EBSCO Health, USA, 2012)	Print	To provide an organized approach for patients with knee OA to decide between medications, joint injections, or knee replacement surgery for managing their pain
Fraenkel et al. (2007)	Name not specified	Computerized survey	To enable patients with knee OA to construct their treatment preferences by asking them to make tradeoffs between competing treatment choices, including pills, creams, injections, and exercise
Gaskin et al. (2020)	Decision tool for knee/joint pain treatment	Print	To provide information to Latina and African American women with knee pain regarding the impact of various surgical and nonsurgical treatment options on quality of life, cost predictions, and work productivity
Hurley et al. (2021)	Name not specified	Video	To provide balanced information about treatment options for knee OA
Ibrahim et al. (2017)	Name not specified (Foundation for Informed Medical Decision Making, USA)	40-min video	To describe both surgical and nonsurgical treatment options, including lifestyle changes, medications, injections, and complementary therapy. The video also discussed the risks, benefits, and efficacy of each treatment option. It also discussed indications for surgery, associated risks, and expected recovery times
Jayakumar et al. (2021)	Name not specified	Computerized, artificial intelligence (AI)-enabled modules	To integrate patient education, preference assessments, and AI-enabled analytics for patients with advanced knee OA considering TKR
Johnson (2021)	Movement is Life shared decision-making tool	Online	To project the personalized impact of various nonsurgical treatment options on the patient’s likely level of pain, activity, and economic productivity at future time points as compared with choosing no intervention
Jones et al. (2022)	Knee osteoarthritis: impact of treatment choices	Online	To allow patients to compare likely outcomes at 1, 5, and 10 years of two nonsurgical or surgical treatment pathways versus no treatment
Mangla et al. (2019)	Arthritis: should I have knee replacement surgery? (Healthwise, 2015)	Printed 15-page brochure	To get facts, compare options, view frequently asked questions, patient stories, and quizzes to encourage patient decision-making regarding knee OA treatment options
Moreton et al. (2022)	The Annalisa decision support tool	Online	To provide an estimate of the value of each of the 20 potential treatment options for patients with knee OA on the basis of user preferences multiplied by the performance of treatments
Reilly et al. (2023)	Name not specified (Emmi Solutions, USA)	Video	To facilitate decision-making and confidence in veterans with knee OA, considering various treatment options
Rivero-Santana et al. (2021)	Name not specified	Computerized	To reduce decisional conflict, increase knowledge of OA disease process and treatment options, and improve satisfaction with the decision-making process for patients with knee OA
Rochon et al. (2014)	Name not specified	Computerized survey	To facilitate decision-making using adaptive conjoint analysis to choose between seven nonsurgical treatment options for patients with knee OA
Sepucha et al. (2019, 2021)	Treatment choices for knee OA (Health Dialog, USA, 2016)	43-min video and 52-page printed booklet	To discuss the concept of SDM, the pathophysiology of arthritis, and available treatment options (both surgical and nonsurgical) for patients with knee OA
Toupin et al. (2016)	Name not specified	Print	To compare the benefits and risks of 13 nonsurgical treatment options for managing knee OA via a step-wise approach and self-assessments. Probabilities of benefits and harms were presented using pictograms
Turnbull et al. (2023)	Name not specified	Online or print	To help patients with knee OA determine their stage of disease and which treatments, both surgical and nonsurgical, are most appropriate for that stage
Van de Velde et al. (2018)	Name not specified	Computerized tool	To facilitate conservative management of patients with knee OA, including pharmacological and nonpharmacological options (e.g., exercise, weight loss)
Van Dijk et al. (2021)	Name not specified	Online tool	To offer patients with knee OA a comparison between operative treatment (TKR) and nonoperative options (e.g., lifestyle advice, painkillers, steroid injections)
Volkmann et al. (2015); Weng et al. (2007)	Treatment choices for knee osteoarthritis (Foundation for Informed Medical Decision Making, USA)	45-min video	To describe both medical and surgical treatment options for knee OA as well as OA pathogenesis and patient testimonials
Volkmann et al. (2015)	Personalized arthritis report on pain and physical function	Printed, 1-page report	To provide patients with knee OA with a visual representation of how their current pain and physical functioning compares with gender- and age-adjusted mean scores for patients who underwent TKR
Washington et al. (2015)	Osteoarthritis of the knee (National Health Service, UK)	Online, self-paced tool	To explain the pathology of knee OA and various treatment choices, as well as differences and similarities between treatment choices
Zheng et al. (2018)	Name not specified	Online, 20-question survey	To provide an individualized estimate of likely patient-level improvement after TKR in pain relief and physical function on the basis of patient-entered demographics, health, comorbidities, and current pain and function

The PDAs came in a variety of formats, with some offering both virtual and print options. At least 19 studies included PDAs with video components [17, 18, 20, 25, 30, 43, 44, 55, 56, 61, 64–66, 68, 70, 71, 74, 79, 81]; 19 studies included PDAs with either an online or computerized interactive component [19, 22, 27, 31, 33, 37, 46–48, 59, 62, 63, 65, 66, 75, 77, 78, 80, 83]; and 23 studies included PDAs that included printed components as either their primary format or as alternative to online versions [17, 18, 20, 25, 27, 32, 35, 39, 50, 55, 56, 61, 64–66, 68, 70, 71, 73–75, 79, 82].

### Outcome measures

Across 69 studies, 29 unique outcome measures assessed various domains of SDM implementation, including decision quality, decisional conflict, SDM process, patient satisfaction, decisional regret, clinical outcomes, and quality of life (Appendix 2).

Decision quality was most often measured using the Decision Quality Instrument (DQI) (15 studies) [16, 17, 19, 20, 25, 46, 55, 61, 62, 64–67, 70, 71], where higher Decision Quality Instrument scores reflect greater patient knowledge and stronger concordance between patients’ values and their chosen treatment. The Decisional Conflict Scale (DCS) was used in 13 studies [16, 17, 22, 30, 32, 33, 53, 62, 65, 70, 71, 78, 79], with lower Decisional Conflict Scale scores indicating reduced uncertainty and greater confidence in decision-making. SDM behaviors were commonly captured using the Shared Decision Making Process (SDMP) scale (7 studies) [26, 62, 64–66, 70, 76] and CollaboRATE (4 studies) [19, 26, 46, 64]. Higher scores on these scales indicate greater patient involvement and perceived collaboration during clinical decision-making. Patient readiness was assessed with the Preparation for Decision Making Scale (PDMS) (four studies) [16, 17, 37, 71] and SURE scale (four studies) [19, 20, 26, 73].

For patient-centered outcomes, functional outcomes were evaluated with the Knee Injury and Osteoarthritis Outcome Score (KOOS) (six studies) [22, 46, 53, 66, 67, 78], with higher scores indicating better knee-related function. Quality of life was measured using EQ-5D (six studies) [24, 55, 58, 64, 66, 67], where higher scores reflect better health-related quality of life. Decisional regret was assessed using the Decision Regret Scale (five studies) [27, 58, 62, 66, 67], with lower scores indicating less regret following treatment decisions.

### Evaluating the benefits of SDM

Among studies evaluating patient outcomes, the most frequently reported benefits of SDM in knee OA included improvements in decision quality, defined by increased patient knowledge or better alignment of decisions with patient goals (19 of 20 studies, 95%) [16, 17, 19, 20, 25, 30, 32, 35, 46, 55, 62, 64–66, 68, 70, 71, 73, 78], and greater satisfaction with their decisions (9 of 9 studies, 100%) [25, 26, 30, 46, 48, 62, 67, 73, 78]. SDM also enhanced patient preparedness for clinical visits (four of five studies, 80%) [25, 37, 70, 80], promoted collaborative decision-making (three of three studies, 100%) [19, 46, 64], and increased patient self-efficacy (two of two studies, 100%) [37, 73]. Patients experienced lower decisional conflict (10 of 13 studies, 77%) [16, 17, 19, 20, 30, 32, 33, 62, 78, 79] and reduced decisional regret (3 of 5 studies, 60%) [26, 27, 67]. Additional reported benefits included lower overall medical costs [18], shorter decision-to-treatment timelines [71], and improved functional outcomes among patients who participated in SDM compared with those who did not (3 of 4 studies, 75%). Regarding treatment choices, most studies found that SDM was associated with a reduced preference for surgery (four of seven studies, 57%) [18, 19, 64, 74], while one study reported no difference, and two observed an increased preference for surgery [20, 46].

Benefits were also noted from the clinicians’ perspectives. All five studies that examined clinician perspectives reported perceived benefits for their patients [23, 25, 29, 36, 73], and one study found that clinicians who engaged in SDM reported increased knowledge and confidence when discussing treatment options [22]. In addition, two studies evaluating the use of PDAs in orthopedic consultations reported high clinician satisfaction, with surgeons noting that most visits (approximately 89%) were of normal duration or shorter, suggesting that integrating SDM did not meaningfully extend visit times [65, 66].

### Factors affecting decision-making

We collected data on demographic, interpersonal, and system-level factors that influence both interest in and success with SDM practices in knee OA. Key considerations in treatment selection, which were subsequently integrated into SDM discussions and PDAs, included information on pain and functional levels (four studies) [38–40, 59], cost (two studies) [39, 59], and potential side effects [59].

Overall, 11 studies examined demographic factors to determine which patient groups are more likely to desire or benefit from SDM. In terms of race/ethnicity, non-white patients reported lower odds of receiving treatment that reflected their post-decision aid choices compared with white patients and had lower expectations about treatment outcomes for both pain and function (two studies) [43, 81]. In addition, patients with knee OA covered by Medicare or Medicaid were less likely to receive treatment aligned with their preferences compared with those with private insurance [43]. Older patients with knee OA reported increased confusion when using online PDA tools and felt overwhelmed by the number of treatment options provided [63]. Notably, another study found that a PDA tool with larger fonts, bigger buttons, and simpler navigation facilitated use among older patients [83]. Patients with lower education or health literacy levels reported lower levels of SDM during clinical encounters, more difficulty using PDA tools, and fewer improvements in decision quality (three studies) [22, 50, 62]. Regarding gender, female patients received less medical information and encouragement to participate in SDM than male patients [21], but following the use of a decision aid, female patients showed greater decision readiness and more accurate treatment expectations compared with male patients who used the aid [79].

Beyond individual demographic factors, interpersonal and cultural contexts also influenced the extent and nature of SDM engagement. One study found that less SDM occurred during consultations when an interpreter was used, as patients were less active participants in the discussion [82]. Interestingly, one study found that patients with knee OA in Saudi Arabia preferred a more paternalistic approach, where they were less involved in the decision-making process [15]. This suggests that cultural attitudes toward patient autonomy can significantly influence preferences for personal involvement in care decisions.

### Barriers to SDM in knee OA

Barriers to implementing SDM in knee OA occurred at multiple levels: system, clinician, and patient.

System-level barriers included limited institutional or organizational support for SDM, lack of integration of PDAs into clinical workflows, and insufficient awareness of available tools at the health system level [29, 75]. In some settings, clinicians reported that the absence of structured processes or incentives to adopt SDM made sustained implementation challenging [75].

Clinician-level barriers included perceived time constraints during visits and concern about the potential to extend patient visit durations (four studies) [17, 24, 29, 36]. In one study, clinicians expressed reluctance to incorporate PDAs into their practice, fearing that even a PDA requiring only 5 min to complete could significantly increase their workload if multiple patients completed it during visits [17]. In addition, clinicians reported challenges in effectively communicating information about treatment options to patients, as well as determining the appropriate amount of information to share (four studies) [36, 39, 49, 63].

From the patients’ perspective, technical issues with online formats were a common challenge (four studies) [22, 55, 63, 75]. One study found that patients were less likely to use a PDA if their pain was more severe, their quality of life was lower, or they were less likely to receive nonsurgical treatment [24]. Patients also expressed confusion about their role in SDM, often believing that decision-making was the clinician’s responsibility and fearing that SDM might shift the burden of decision-making onto them (three studies) [15, 30, 36]. To overcome these barriers, patients generally preferred shorter PDAs over longer ones (two studies) [65, 66] and preferred using the tool before meeting with their clinician (two studies) [39, 56].

## Discussion

This scoping review synthesized current literature on SDM in knee OA, highlighting potential benefits, consideration factors, and barriers to implementation. As a scoping review, this work is intended to provide a descriptive overview of the existing literature and to generate hypotheses, rather than to assess comparative effectiveness or establish causal relationships. It is also important to note that a subset of the included studies enrolled mixed osteoarthritis populations (most commonly involving both the knee and hip), which constrains knee-specific interpretation of some synthesized findings.

Collectively, the included studies suggest that SDM, often facilitated by PDAs, may hold promise for improving decision quality, reducing decisional conflict, and enhancing patient satisfaction among patients with knee OA. Factors that influence SDM use include patients’ pain and functionality levels, patient demographics, including socioeconomic status, age, and race, and the ease of use of the PDAs. Challenges remain in optimizing SDM practices across diverse patient populations and clinical settings, including perceived time constraints, lack of awareness of existing PDAs, and confusion about the role of SDM in patient–clinician discussions [29, 36, 75].

SDM has been associated with medical outcome domains such as decision quality, decision conflict, and treatment satisfaction, which may have downstream effects on clinical outcomes [6, 7]. For example, patients engaging in SDM have been reported to be more proactive in their knee OA management by better adhering to exercise programs or conservative therapies [7, 8]. While there is limited evidence linking SDM to improvement in pain and function, these speculative pathways highlight why SDM can have a broader clinical relevance that goes beyond the decision-making process itself [12, 67]. Knee OA serves as an appropriate context for the evaluation of SDM, as this condition is widely considered preference-sensitive with multiple acceptable treatment options [12]. As a result, it can be challenging for patients to navigate through options that differ in their risks, benefits, and strength of clinical recommendations. These individualized trade-offs highlight the relevance of SDM in promoting treatment decisions that are reflective of the patient’s core values [6].

A promising way to implement SDM into practice is through the use of PDAs [7]. However, we observed wide variability and a lack of consistency among the PDAs that were utilized by the studies included. In most cases, the PDAs are also not publicly available for comparison, making it difficult to assess similarities and differences among tools. Furthermore, many different mediums are utilized to present tools (e.g., online video, DVD, booklet, computerized survey) with a lack of consensus or evidence as to which format is preferred by patients. Without consistently naming and describing tools in the literature, it is difficult to discern which tools have been repeatedly studied or draw any conclusions about one individual tool being superior to another. Nonetheless, most studies that utilized a PDA, of any format, to uniformly implement SDM reported at least neutral or positive outcomes overall [10]. Therefore, it may be worthwhile to make PDAs that are publicly accessible to easily replicate study designs and compare results in other populations.

From a clinical standpoint, both the variability and the lack of consensus over the optimal PDA make it difficult for clinicians to implement PDA in their practice [29, 36]. Nevertheless, clinicians should still critically appraise their PDA of choice before use to ensure accuracy, appropriateness, and consistence with the current evidence of care [6, 9]. Moreover, clinicians should identify PDAs that are culturally and linguistically appropriate for the specific patient population being served [82]. Identifying the barriers to the SDM process, as discussed above, is essential to extrapolate maximal benefits from PDAs for patients in need [29, 36]. In that regard, future studies aimed toward establishing a consensus on the optimal form of PDA among clinicians managing knee OA can be extremely beneficial. Developing a standardized PDA for knee OA that is consistent with current clinical guidelines could potentially help further minimize PDA variability and improve accessibility [3, 7]. Moreover, it is important to recognize the disease-specific challenges that can complicate SDM implementation. Given the complex course of knee OA, patients may have skepticism about the effectiveness of conservative treatments and misconceptions about surgery. Physicians may also find it challenging to simplify the complex management of knee OA within a limited visit time. Despite these difficulties, facilitators such as a standardized SDM tool, a multidisciplinary care team, and a structured clinic workflow can act to counteract knee OA-specific barriers [29, 75].

Beyond PDA-related considerations, broader determinants of health, such as culture, gender, and healthcare system structure, may influence the feasibility and uptake of SDM in knee OA as well [15, 43, 82]. Cultural values surrounding pain and treatment methodologies can impact a patient’s trust or their willingness to engage in the proposed medical plan [15, 82]. Gender-related factors, including gender-specific medical conditions and differences in healthcare experiences, can also impact SDM engagement [21, 79]. Furthermore, systemic factors such as access to educational resources, insurance coverage, and socioeconomic status can exacerbate the inaccessibility of SDM [22, 43]. Recognizing these intersecting determinants of health is important for establishing and improving the applicability of SDM for patients with knee OA in the future.

Overall, the results of our scoping review suggest several actionable steps for improving SDM in knee OA. First, developing standardized, user-friendly PDAs that cater to diverse patient populations (such as older adults and those with lower health literacy) could enhance the reach and effectiveness of SDM [7, 83]. Second, integrating SDM into the clinical workflow in ways that minimize the time burden on clinicians, such as introducing PDAs prior to consultations, could help overcome clinician resistance [39, 56]. Finally, addressing clinician education and system-level support for SDM could aid in its successful implementation [29, 75].

## Limitations

Several limitations to this review should be acknowledged. As described previously, many studies did not provide detailed descriptions of the PDAs utilized, making assessing their comparability or effectiveness difficult. Secondly, we suspect that there are more than 44 PDAs available for knee OA, as PDAs may not always be shared publicly or may not have been published in editorials. Moreover, even for the PDAs that were published in studies, the heterogeneity of study designs and outcomes complicates efforts to synthesize findings across different contexts and populations. Several studies included patients with both knee OA and other-joint OA but did not separately report results. We opted to include these studies to maximize coverage of knee patients, acknowledging that some outcomes may therefore reflect mixed-joint populations and lack specificity to knee patients. Finally, because the studies included had varied methodologies, our use of frequency counts allowed us to capture the prevalence of findings but also limited our ability to quantitatively compare the significance of benefits, drawbacks, and other factors relative to each other.

## Future research

Our findings highlight the need for future research to explore the existence and accessibility of PDA tools, as well as their comparative effectiveness in assisting management decisions. Furthermore, standardized outcome measures and a consistent framework for evaluating PDAs would enhance the comparability and generalizability of findings. Future studies should also evaluate the effectiveness of PDAs among diverse patient populations, considering variations in age, severity of OA, cultural backgrounds, and health literacy. More specifically, future studies should examine the use of culturally tailored PDAs and its impact on reducing cultural misinterpretations and the mitigation of linguistic and cultural barriers. Research that explores the long-term impact of PDAs on patient outcomes and satisfaction, as well as their influence on SDM processes in clinical settings, could similarly provide valuable insights. Finally, studies examining the integration of PDAs into digital platforms could expand access and support patient engagement in a variety of care environments. By delivering PDAs via various digital formats, patients can conveniently review the available treatments at their leisure outside of a medical setting, ensuring basic comprehension of the clinical reasoning behind conservative, pharmacologic, and surgical approaches while maintaining patient autonomy in decision-making.

## Conclusions

This scoping review provided an overview of current evidence on SDM practices in knee OA management. Our findings suggest that incorporating SDM through the use of PDAs has been commonly associated in the literature with improved active engagement, decision quality, and patient satisfaction, as well as reductions in decisional conflict and regret. However, variability in SDM implementation and inconsistency in outcome measures underscore the need for further research to evaluate the comparative effectiveness of decision aids in clinical practice. Advancing SDM practices may help empower patients to make informed choices, ultimately improving health outcomes and quality of life for those affected by knee OA.

## Supplementary Information

Supplementary Material 1.

Supplementary Material 2.

## Data Availability

The datasets analyzed during the current study are available from the corresponding author on reasonable request.
